# Autonomic Nervous System Control in Male and Female Elite Soccer Players: Importance of Different Training Routines and Perceived Stress

**DOI:** 10.3390/jcdd12040150

**Published:** 2025-04-10

**Authors:** Gianluigi Oggionni, Eleonora Pagani, Jacopo Rizzardini, Margherita Rigillo, Luca Giovanelli, Mara Malacarne, Nuno Loureiro, Júlia Machado Ribeiro, Piero Volpi, Massimo Pagani, Daniela Lucini

**Affiliations:** 1 IRCCS Istituto Auxologico Italiano, Exercise Medicine Unit, 20135 Milan, Italy; g.oggionni@auxologico.it (G.O.); massimo.paganiz@gmail.com (M.P.); 2 Department of Psychology, Catholic University of the Sacred Hearth, 20123 Milan, Italy; eleonora.pagani@unicatt.it; 3 BIOMETRA Department, University of Milan, 20129 Milan, Italy; jacopo.rizzardini@unimi.it (J.R.); margherita.rigillo@unimi.it (M.R.); luca.giovanelli@unimi.it (L.G.); mara.malacarne@unimi.it (M.M.); 4 Sporting Clube de Portugal, Medical and Performance Department, 2890-529 Lisbon, Portugal; nmloureiro@sporting.pt (N.L.); jribeiro@sporting.pt (J.M.R.); 5 IRCCS Humanitas Research Hospital, 20089 Rozzano, Italy; piero.volpi@humanitas.it

**Keywords:** sport psychology, HRV, heart rate variability, autonomic nervous system, sympathetic activity, parasympathetic activity, stress, football

## Abstract

The assessment of cardiac autonomic regulation (CAR) with non-invasive techniques, such as heart rate variability (HRV), might be of practical interest in elite sports, considering its importance in determining training. We studied 117 soccer players (74 male and 43 female) from three First Division European soccer teams. We used a ranked Autonomic Nervous System Index (ANSI, resulting from the combination of multivariate statistical methodologies applied to HRV-derived indices) to assess CAR. We hypothesized that ANSI might differentiate playing positions, considering goalkeepers, defenders, midfielders, and forwards. We also assessed the perception of somatic symptoms and stress. We observed in male athletes that ANSI was significantly greater in males as compared to females (*p* < 0.001), being higher in midfielders and defenders (*p* = 0.035), who usually sustain the greatest external load. Interestingly, this result was not observed in female athletes, who, instead, reported a significantly higher perception of somatic symptoms (*p* = 0.018) and stress (*p* = 0.049), the latter being particularly high in midfielders and forwards (*p* = 0.045). This approach might represent a convenient model to study the effects of long-term physical exercise on CAR in soccer, even to unveil possible differences due to sex, different playing positions characterized by distinct exercise routines, or stress perception.

## 1. Introduction

Soccer is currently the world’s most popular sport [1], and its appeal is expanding beyond just males to include the female population as well. From Europe, where it is known as football, to East Asia, Africa, and the Americas, over 40% of the global population expresses an interest in soccer [2]. This widespread popularity presents a unique opportunity to engage in physical exercise, promoting well-being, preventing chronic non-communicable diseases, and encouraging socialization and healthy habits, particularly among young people. The role of exercise as a tool to promote health is well established [3,4]; it may improve many control mechanisms such as endocrine, immunological, and autonomic ones. In particular, the effect of physical activity (specifically aerobic training) on cardiac autonomic control (CAR) explains, albeit in part, the important reduction in cardiometabolic and oncological mortality and the stress management role of exercise and the improved well-being [5,6,7,8]; conditions which motivate all the main health institutions to insert it in preventive guidelines [3,4,9] and policies to foster health [3,10]. Conversely, the intense execution of exercise requires an increase in sympathetic activity, leading to a rise in heart rate, blood pressure, and myocardial contractility in the short term, as well as potential cardiovascular adaptations in the long term [11,12]. These conditions, close to other mechanisms, may increase the risk of acute cardiac events, such as arrhythmias or ischemic damage, particularly in subjects with chronic cardiometabolic disease, or in subjects with a genetic predisposition for arrhythmias performing exercise at vigorous intensity, particularly if associated with the stress of competition, like in some elite athletes [13,14]. Structural cardiac remodeling, specific to the exercise loads, may determine an increase in myocardial wall and chamber thickness; moreover, the increases in myocardial demand and adrenergic output (associated with exercise) may induce an arrhythmogenic state or ischemia, precipitating sudden cardiac arrest [15,16].

Hence, the autonomic nervous system may play a double role: on the one side, aerobic training is a fundamental tool to foster health and prevent chronic diseases; on the other side, the acute execution of a maximal dose of exercise may trigger acute, even fatal, cardiac events.

The possibility of studying CAR in clinical and sports settings is gaining greater interest, especially employing the study of RR interval variability, usually called heart rate variability (HRV), a simple, economical, and non-invasive technique that represents nowadays the de facto standard method to assess CAR in clinical settings. Nevertheless, the large-scale use of this non-invasive technique presents some critical methodological and interpretation issues. To address part of these criticalities [17,18] (in particular the difficulties in interpreting the wide number of variables furnished by the analysis and the age and sex bias) we introduced a novel, composite, ranked Autonomic Nervous System Index (ANSI) [17,19,20] which represents a single composite percentile-ranked proxy of autonomic balance, whereby higher values indicate better autonomic control [17]. ANSI is, by design, free of age and sex bias, and is built considering only variables derived from heart rate variability, without the need to record systolic arterial pressure. This index was capable of showing the effect of aerobic training both in patients [21] and athletes [22,23] and of characterizing different body mass compositions in both inpatients and athletes [24,25], differentiating the effects of training in elite basket players [22] and unveiling differences in CAR in Olympic athletes characterized by different training loads [26] (using a more sophisticated version of this index which considers also variables derived from exercise stress tests). In a previous paper on soccer players, we observed that ANSI was greater compared to similar age controls and was highest in midfielders and defenders, who usually sustain the greatest external load during competitions [23].

In studying cardiac autonomic control, it may be of note to consider also the important effect of stress on it. Stressors widely impact all body control mechanisms [27,28], in particular, the autonomic nervous system, determining both acute and chronic effects, ranging from the presence of somatic symptoms [27,28] not explained by other diseases to the increased risk of cardiometabolic, oncological, and psychiatric diseases, to reduced well-being and performance [29,30,31,32,33,34]. Stress can greatly affect elite athletes by leading to overtraining syndrome and impaired CAR, which is crucial for their performance and recovery [35].

In the present paper, we explore the hypothesis that the use of a composite ranked Autonomic Nervous System Index (ANSI), which represents a single composite percentile-ranked proxy of autonomic balance, was capable of unravelling possible sex differences in CAR in a population of male and female elite soccer players, also considering the effects of different training loads (which characterized different player positions) and a possible role of stress perception.

## 2. Materials and Methods

This observational, retrospective study is part of an ongoing series of investigations focusing on the use of autonomic nervous system evaluation to monitor physical training in elite athletes. These data refer to a group of 117 elite soccer players from three First Division European soccer teams, considering athletes from both the First Teams (N = 63) and B Teams (N = 54), both males (N = 74) and females (N = 43). The health of the athletes was ensured by their team doctor and confirmed by history and physical examination. Evaluations were performed in January–March (for one Italian First Division team) and in June–August (for the other Italian First Division team and the Portuguese First Division team). All subjects had provided informed consent at the time of the visit, and they were informed and agreed that their anonymized data could be used for scientific projects. The protocol of this study followed the principles of the Declaration of Helsinki and Title 45, US Code of Federal Regulations, Part 46, Protection of Human Subjects, Revised 13 November 2001, effective 13 December 2001 and approved by the Ethics Committee of the University of Milan on 18 April 2023 and the Independent Ethics Committee of Humanitas Research Hospital (Rozzano, Italy) on 13 October 2015.

Evaluations considered:

### 2.1. Clinical Evaluation

Clinical assessment: anthropometric (height, weight) and hemodynamic parameters (systolic/diastolic arterial pressure and resting heart rate).

### 2.2. Study of the Cardiac Autonomic Control (CAR)

Cardiac autonomic regulation (CAR). Recordings were performed in an ad hoc-equipped room located near the football training fields. ECG and respiratory activity (piezoelectric belt) were continuously recorded over a minimum 5 min period with a two-way radiotelemetry system (Marazza, Monza, Italy) after a preliminary 10 min rest period in the supine position and during stand-up for 5 min. Data were acquired with a PC at 250 samples/s using a custom-built software tool (HeartScope, version 2.0) which automatically provided a series of indices describing HRV in the time domain: RR interval (in ms) and RR interval variability (assessed as total power, i.e., variance, in ms^2^), taken as simple classifiers typical of vagal control [17,20,26] and in the frequency domain: autoregressive spectral components both in the low frequency (LF, center frequency ≈ 0.1 Hz) and in the high frequency (HF, centered with respiration, ≈0.25 Hz), assessed in ms^2^ as well as in normalized units (nu). To simplify the clinical interpretation of multitudes of HRV variables jointly considered, we developed a unitary autonomic index (Autonomic Nervous System Index, ANSI) [17,20,24] as a proxy for CAR. This index was built based on the main findings derived from factor analysis applied to HRV variables, whereby RR, RR interval variance, and ΔRR LFnu resulted in being highly representative of the cardiac autonomic information (considering amplitude and oscillatory code modalities). The procedure to construct ANSI is the following: First, to obtain variables adjusted for age and sex effects [36], the percentile rank (PR) transformation is applied to, respectively, RR, RR interval variance, and ΔRR LFnu within each age-by-sex class. Second, to obtain a unitary index, a radar plot (with a triangle shape) is built for each subject using the values of the three PR-transformed variables; then, the areas of the individual triangles are computed. Third, to have a normalized index, the PR transformation is applied again to the individual triangle areas. This way, the obtained ANSI can be regarded as a composite normalized indicator, free of sex and age bias, ranging from 0 to 100, where higher values denote better autonomic control [17]. Arterial pressure was assessed using electronic sphygmomanometers (A&D Medical, Model UA-767 plus30, Tokyo, Japan).

### 2.3. Evaluation of Stress Perception

Stress and somatic symptom perception were assessed in 98 athletes (males = 55, females = 43) using a self-administered questionnaire [37,38] providing nominal self-rated scales (higher values indicate higher degrees of symptoms) that focused on (i) the appraisal of the overall stress and fatigue perception by evaluation scales with integer scores from 0 (‘no perception’) to 10 (‘highest perception’) for each measure; (ii) the Short-Subjective Stress-related Somatic Symptoms Questionnaire (4S-Q), inquiring about four somatic symptoms accounting for the majority of somatic complaints. For scoring purposes, each response was coded from 0 (‘no feeling’) to 10 (‘a strong feeling’); thus, the total score ranged from 0 to 40. We asked the athletes to fill out the questionnaire immediately after the clinical assessment and CAR evaluation.

### 2.4. Statistics

Descriptive statistics of the studied variables were computed as mean ± SD. Differences between male and female athletes were assessed with an independent sample *T*-test, indicating Effect size (Cohen’s d). The relatively small number of subjects required consideration, keeping in mind the feasibility of the study. Although it is not a barrier in itself, it calls, however, for careful interpretation, avoiding generalizations and preferring methods (such as Cohen’s d) that are not relatively affected by the number of subjects and focusing on the magnitude (small = 0.2; medium = 0.5; large = 0.8; very large = 1.3), not on the probability of an effect [39].

Differences between groups (goalkeepers, defenders, midfielders, and forwards) were assessed with 1WAYANOVA followed by individual contrasts; *p* < 0.05 was set as the level of significance. Statistical analysis was performed using SPSS version 29 (IBM Corp., Armonk, NY, USA). *p* values < 0.05 were considered statistically significant.

## 3. Results

### 3.1. Cardiac Autonomic Regulation (CAR) and Anthropometrics Data

Table 1 reports data considering the overall population. As expected, males were characterized by higher body weight, height, BMI, and systolic and diastolic arterial pressure. In this population, males presented a higher RR VAR (variance of RR interval variability), higher RR HFnu (the high-frequency component of RR interval variability, marker of prevalent vagal modulation), and higher ANSI (a unitary autonomic index considered as a proxy for overall autonomic nervous system control [17]).

**Figure 1:**
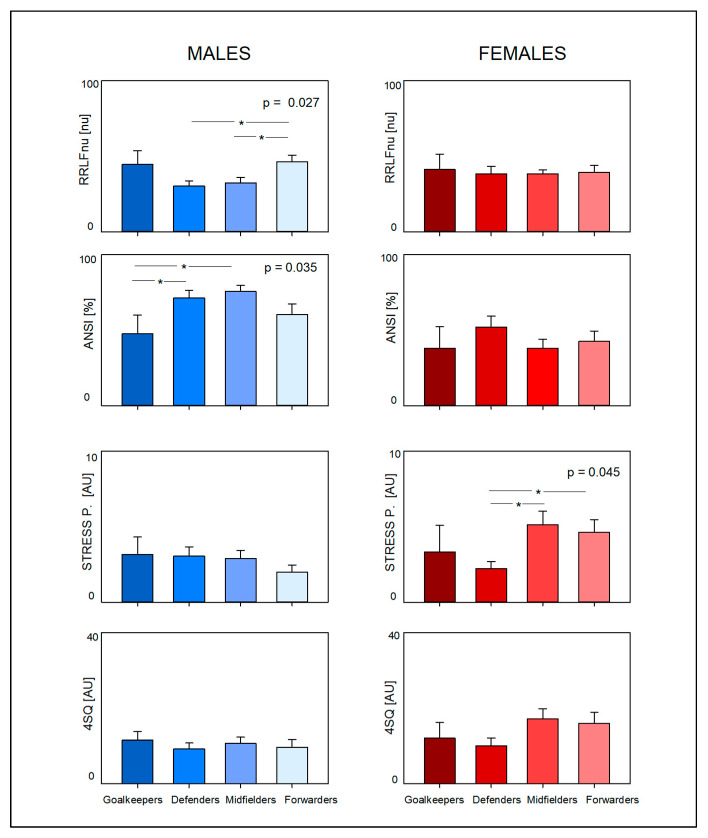
Main autonomic and stress perception Proxies in Male (**left** panels) and Female (**right** panels) soccer players, considering playing position. RRLFnu = low-frequency component of RR variability expressed in normalized units; ANSI = Autonomic Nervous System Index; P = perception; AU = arbitrary units. 4SQ = short somatic symptoms stress-related questionnaire. * significant contrast.

Considering the different playing positions (see Table 2 and Table 3 and Figure 1), data showed that in male soccer players, it was possible to unveil differences regarding anthropometrics and CAR. Specifically, male goalkeepers were notably taller and heavier than players in other positions, while midfielders and forwards demonstrated lower body weights compared to defenders. It is particularly noteworthy to observe that the LF RRnu (the low-frequency component of RR interval variability, marker of prevalent sympathetic modulation) was significantly different (*p* = 0.027) in the overall male group, being greater in forwards as compared to defenders and midfielders, and vice versa for the HF RRnu (the high-frequency component of RR interval variability, marker of prevalent vagal modulation). Moreover, the ANSI (a unitary index, overall proxy of CAR) was significantly different (*p* = 0.035) in the overall male group, being greater in defenders and midfielders (who usually sustain the greatest external load) as compared to the other playing positions. In contrast, no differences were observed in the female group.

### 3.2. Stress and Somatic Symptoms Perception Data (See Table 1, Table 2, Table 3 and Table 4 and Figure 1)

The results of the stress and somatic perception assessments were contrary to those of the CAR assessment. Significant differences were observed solely within the female group, where female athletes exhibited a heightened perception of stress (*p* = 0.049) and somatic symptoms (*p* = 0.018) as compared to males. Specifically, female midfielders and forwards reported a higher perception of stress compared to defenders (*p* = 0.045). Additionally, while the perception of somatic symptoms was also greater among female midfielders and forwards, it did not reach statistical significance.

**Table 4:** Individual anthropometrics and autonomic and stress perception Proxies according to playing position, considering female soccer players.

Variable		Goalkeepers	Defenders	Midfielders	Forwards	*p*
N		3	14	15	11	
Age	[yrs]	22.33 ± 5.13	20.64 ± 5.51	19.33 ± 4.40	20.82 ± 6.29	0.782
Weight	[kg]	63.67 ± 9.02	57.04 ± 5.37	54.70 ± 7.86	55.91 ± 8.34	0.288
Height	[cm]	171 ± 7	166 ± 5	163 ± 7	164 ± 5	0.152
BMI	[kg/m^2^]	25.49 ± 2.96	24.68 ± 1.49	24.70 ± 2.91	24.63 ± 2.69	0.928
SAP	[mmHg]	108.33 ± 14.43	109.14 ± 11.39	109.13 ± 10.60	108.55 ± 7.83	0.998
DAP	[mmHg]	68.33 ± 14.43	62.79 ± 9.17	63.80 ± 9.91	63.09 ± 8.38	0.832
HR	[beat/min]	61.43 ± 3.94	59.42 ± 7.92	63.14 ± 9.47	64.94 ± 10.10	0.472
RR	[ms]	979 ± 65	1027± 140	969 ± 136	942 ± 135	0.460
RR VAR	[ms^2^]	9611 ± 12,562	5982 ± 2744	7213 ± 5687	5604 ± 2795	0.572
RR LFa	[ms^2^]	2172 ± 2147	1699 ± 1516	1950 ± 1809	1474 ± 1018	0.844
RR HFa	[ms^2^]	4502 ± 6802	2408 ± 1769	2971 ± 2590	2142 ± 1697	0.510
RR LFnu	[nu]	41.34 ± 17.63	38.26 ± 18.67	38.40 ± 9.70	39.55 ± 14.72	0.986
RR HFnu	[nu]	46.46 ± 26.04	52.52 ± 19.27	52.95 ± 15.94	51.58 ± 15.60	0.949
RR LF/HF		1.55 ± 1.76	1.71 ± 3.81	0.89 ± 0.61	1.00 ± 0.95	0.780
ANSI	[%]	38.18 ± 24.43	51.95 ± 27.12	37.95 ± 23.90	42.61 ± 22.83	0.479
Stress P score	[AU]	3.33 ± 3.06	2.21 ± 1.81	5.13 ± 3.48 *	4.64 ± 2.77 *	**0.045**
Fatigue P score	[AU]	4.00 ± 2.65	2.79 ± 1.93	4.53 ± 2.67	3.00 ± 2032	0.205
4SQ score	[AU]	12.00 ± 7.21	10.00 ± 7.64	17.20 ± 10.25	16.00 ± 9.52	0.176

Data are expressed as mean ± SD; *p* = overall significance by 1WAYANOVA, followed by individual contrasts. Significant (*p* < 0.05) individual contrasts: * vs. goalkeepers. Abbreviations: RR = RR interval; HR = heart rate; V = variability; RR VAR = Total Power of RR interval variability (variance); LF = low-frequency component of RR variability; a = absolute value; HF = high-frequency component of RR variability; nu= normalized unit; SAP = systolic arterial pressure; DAP = diastolic arterial pressure; ANSI = Autonomic Nervous System Index; P = perception; AU = arbitrary units; 4SQ = short somatic symptoms stress-related questionnaire. Significant values are written in bold.

## 4. Discussion

In this observational study, we observed that the use of a composite ranked Autonomic Nervous System Index (ANSI), which represents a single composite percentile-ranked proxy of autonomic balance, was capable of showing sex differences in CAR in a population of male and female elite soccer players, considering also the effects of different training loads (which characterized different player positions) and a possible role of stress perception. Male athletes of a First Division European soccer team exhibited a significantly greater ANSI compared to their female counterparts. Notably, female athletes reported a significantly higher perception of stress and somatic symptoms than males. Furthermore, when examining different playing positions, we found that ANSI was significantly higher in male midfielders and defenders, who typically endure the greatest aerobic external load during competitions. In contrast, female midfielders and forwards reported a significantly higher perception of stress compared to other female athletes.

### 4.1. Cardiac Autonomic Regulation (CAR)

Long-term endurance training is generally associated with cardiac neural remodeling, which fosters cardioprotective vagal mechanisms [40], whereas resistance training appears to have a lesser impact on vagal regulation, leading to a less consistent reduction in cardiovascular risk. The assessment of CAR in sports is becoming increasingly popular [41,42]. CAR plays an essential role in the complex chain of mechanisms responsible for athletic performance, and optimal autonomic regulation is a fundamental component for successful training in elite athletes. Specifically, intermediate levels of training loads are associated with signs of increased vagal drive [43,44,45,46], while, as the time of competition and training volumes intensify, a more complex picture, possibly including sympathetic activation, might be observed [26,45,46]. Changes in autonomic balance may unravel the quality of training at various loads and possibly foretell overreaching and overtraining [35,47,48].

Spectral analysis of heart rate variability (HRV) is a simple, non-invasive technique used to assess sympatho-vagal regulation of the heart, it is nowadays considered the “de facto” methodology to study non-invasively CAR and it is also widely employed in sports [18,19,40,41,42,43,45,46,47,49]. Nevertheless, the numerous recent studies on the autonomic nervous system (ANS) and exercise present an inconsistent picture due to a lack of consensus on methodologies and the interpretation of metrics used to extract underlying information [18,19,50].

HRV conveys embedded autonomic information across various indices and multiple domains, employing different coding modalities that may highlight specific physiological aspects with varying degrees of accuracy [51]. Thus, careful mathematical manipulation of experimental data may aid in highlighting specific elements of CAR [52]. In interpreting the results of this methodology, it may be useful to conclude that the model of paired (vagal and sympathetic) antagonistic innervation characterizing peripheral cardiac regulation, and its connection with central autonomic structures, implies a fundamentally “unbroken” unitary nature of neural visceral regulation [53], which may be better described by a unitary comprehensive metric rather than multiple partial indices. Information from various autonomic variables may be difficult to compare when employing direct measures [54] because of different units and directional changes related to excitatory or inhibitory physiological stimuli. Indeed, HRV analysis furnishes several indices that are taken as proxies of vagal (inhibitory) or sympathetic (excitatory) neural modulation in several conditions, from the domains of rest, standing up, and exercise [17,18,19,52]. Considering the fundamentally unitary nature of visceral neural regulation [53], the idea of simplifying HRV analysis by integrating multiple indices into a unitary autonomic proxy [17,26] might be of practical value. Among issues to consider in the interpretation of HRV-derived variables, the roles of age and sex need to be considered [17,26,36,55,56,57]. To address, albeit in part, the criticalities in HRV interpretation, we recently introduced a novel, composite, ranked Autonomic Nervous System Index (ANSI) [17], which represents a single composite percentile-ranked proxy of autonomic balance, whereby higher values indicate better autonomic control [17]. ANSI is intentionally designed to be free from age and gender bias and demonstrates a strong correlation with cardiac baroreflex gain [20]. It is built by integrating the information carried by highly representative components of the cardiac autonomic information (RR Mean, RR VAR, and stand-rest difference in RR LFnu), as indicated by factor analysis, considering a multitude of HRV indices. Moreover, it provides a unitary proxy of CAR, expressed as a percentile rank against a reference benchmark population, offering a standardized measure that also encompasses more complex information, allowing, through coherent integration, for the detection of minor changes in multiple variables [17]. The 0–100 ranking facilitates simple comparisons between individuals, conditions, or time periods, and it is easy to understand.

In a previous study involving male soccer players, we found that ANSI was higher compared to age-matched controls, with the highest levels observed in midfielders and defenders, who typically endure the greatest external loads during competitions [23]. In this paper, we confirmed our previous findings with a larger study population and expanded upon this observation by noting that the enhanced autonomic control seen in male midfielders and defenders was not present in female athletes. This discrepancy may be attributed to potential differences in specific training regimens or the varying technical aspects of elite male and female soccer players [58,59,60,61]. There are technical, tactical, and physical differences between men’s and women’s games [62,63,64]. For example, male players cover greater distances in high-intensity running compared to female players during match play [62]. Additionally, the differences between female and male athletes are not solely attributed to genetic factors; they are also shaped by the levels of selection, training, and competition [65]. For instance, McFadden [60] showed that the distance covered in the higher speed zones across practices and games was found to be greater in men than women; Gioldasis [66] reported that female players performed significantly lower scores in most of the technical skills analyzed in the study; using machine learning technique, Garnica-Caparrós [67] demonstrated differences between female and male performances, while Pappalardo [68] revealed that differences in several technical features considering male and female soccer. It is generally believed that male football is technically more advanced, although there is limited evidence to support this claim [62]. To help female athletes benefit from the higher physical and technical demands typically experienced by their male counterparts, some training routines encourage women to occasionally train alongside men [62].

In this paper, we also reported a significant difference in autonomic control between male and female soccer athletes when considering the overall group. Male soccer players appear to have a more favourable autonomic profile. These data appear to contrast with the established observation [36,55,56,57] that female subjects are characterized by a prevalent parasympathetic control; nevertheless, we have to consider the paramount influence of endurance training [43,44,45,46] and stress in modulating CAR [27,37,38,69]. As discussed in the previous paragraphs, certain technical, tactical, and physical differences between men’s and women’s games may influence exercise training and, consequently, the autonomic profile. The greater males’ cardiopulmonary fitness [63,70] may play an important role in this regard.

### 4.2. Stress Perception

In this paper, we also described that female athletes are characterized by a higher perception of somatic symptoms and stress; the latter one being particularly elevated in female midfielders and forwards, those athletes who are more expected to score goals. This fact might play a key role in affecting autonomic control and may in part explain the worst autonomic control that characterized females in this study. Sports psychology is growing progressively due to its potential role in helping athletes manage stress, improve performance [71], and reduce the occurrence of injuries. Acute stress may be a useful adaptive response; nevertheless, it can affect performance [72] and health [73,74]; chronic stress may represent one of the most important factors that may negatively impact the health and performance of athletes [32,75,76,77]. Both acute and chronic stress are characterized by CAR impairment [38,69,78], and stress management techniques, such as mindfulness and relaxation training, can improve autonomic control [38,79]. The study of CAR in subjects experiencing stress conditions, particularly in sports settings, may represent a challenging issue, considering that the employment of any invasive, or perceived as dangerous, techniques may per se be a stressor; moreover, simply considering variables, such as heart rate, arterial pressure, and skin conduction, that are under autonomic nervous system control but are not “measure” of this mechanisms, may be considered misleading and not sufficient to investigate ANS accurately. Notably, autoregressive HRV may overcome these issues and represent a convenient, albeit indirect, method to study the effects of stressors on the autonomic nervous system and the efficacy of stress management intervention [79,80,81,82,83,84].

In this paper, we assessed stress and somatic symptoms perceptions simply using a self-administered questionnaire [37,38], providing nominal self-rated scales (higher values indicate higher degrees of stress); this methodology is very simple to apply and was employed by our group in many studies, showing that scores significantly correlate with HRV-derived parameters [38]; moreover, it was capable of differentiating rowing athletes who received a medal in the world championship from athletes who did not [85] and to differentiate athletes (competing in specialities with low or medium intensity activity levels) who received a medal in the Rio Olympic Games from athletes who did not [26]. Interestingly, we found that stress perception was particularly high among female midfielders and forwards. A similar pattern was observed across different playing roles regarding the perception of somatic symptoms, although this was not statistically significant (see Figure 1).

### 4.3. Limitation

We have to acknowledge some limitations:

Firstly, the research did not measure any technical or tactical variables that may elucidate differences between male and female games. Secondly, data collection occurred at two different seasonal moments: January for one Italian First Division team and June–August for others. This variance may affect training outcomes, though it is crucial to note that the athletes were part of three distinct teams, each with varying training routines and timings that may mitigate seasonal effects. Thirdly, the sample size of enrolled athletes is relatively small, particularly among goalkeepers; however, all participants are elite players from three major First Division European teams. We did not conduct a sample size estimation and used a convenience sampling strategy to select our study population; this strategy, albeit not ideal, may be the only solution in conditions, such as in this study, where the small number of goalkeepers does not depend on researcher choices but on soccer team composition. We attempt to control any other bias and disclose methods and findings transparently [86]. We are aware that the results of this study may not be generalized, but they might be employed to generate hypotheses that can be tested in greater depth in future research.

Lastly, autonomic activity was not directly measured, relying instead on indirect estimates derived from beat-by-beat RR interval analysis. Despite these limitations, the approach allows for the integration of multiple variables into a single composite index, facilitating comparisons while accounting for potential gender and age biases.

## 5. Conclusions

We observed that male elite soccer players present significantly greater ANSI, compared to their female counterparts, who, instead, are characterized by higher perception of stress and somatic symptoms. The use of ANSI, which overcomes some drawbacks of heart rate variability interpretation, such as age and sex bias, may corroborate the use of heart rate variability to study cardiac autonomic control. From a practical clinical perspective, reducing a multidimensional phenomenon to a unitary, combined, 0–100 ranked proxy of CAR might further contribute to the introduction of autonomic evaluation in sports training. These data, however, need to be tested on a large population in future research.

## Figures and Tables

**Table 1:** Individual anthropometrics, autonomic, and stress perception Proxies in all male and female soccer players.

Variable		All	Males	Females	*p*	Cohen’s d
N		117	74	43		
Age	[yrs]	20.54 ± 4.36	20.65 ± 3.81	20.35 ± 5.22	0.74	−0.07
Weight	[kg]	68.63 ± 11.54	75.74 ± 6.39	56.40 ± 7.41 *	**0.00**	**−2.85**
Height	[cm]	175 ± 0.11	182 ± 0.07	165 ± 50.06 *	**0.00**	**−2.63**
BMI	[Kg/m^2^]	25.23 ± 2.08	25.52 ± 1.84	24.73 ± 2.39 *	**0.03**	0.48
SAP	[mmHg]	119.65 ± 15.99	125.88 ± 15.5	108.93 ± 10.11 *	**0.00**	**−1.23**
DAP	[mmHg]	67.91 ± 9.08	70.41 ± 7.97	63.60 ± 9.34 *	**0.00**	**−0.8**
HR	[beat/min]	56.41 ± 10.33	53.00 ± 9.59	62.27 ± 8.90 *	**0.00**	**0.99**
RR	[ms]	1098 ± 198	1166 ± 198	981 ± 134 *	**0.00**	**−1.04**
RR VAR	[ms^2^]	8839 ± 12,474	10,159 ± 15,128	6568 ± 4864	0.06	−0.29
RR LFa	[ms^2^]	1975 ± 2138	2098 ± 2427	1762 ± 1522	0.36	−0.16
RR HFa	[ms^2^]	4078 ± 8187	4889 ± 10,049	2682 ± 2542	0.08	−0.27
RR LFnu	[nu]	36.88 ± 17.56	35.74 ± 19.17	38.85 ± 14.37	0.32	0.18
RR HFnu	[nu]	57.39 ± 19.41	60.53 ± 20.08	52.01 ± 17.10 *	**0.02**	−0.45
RR LF/HF		1.04 ± 1.70	0.93 ± 1.70	1.23 ± 2.26	0.43	0.18
ANSI	[%]	59.34 ± 27.97	68.42 ± 25.81	43.71 ± 24.65 *	**0.00**	**−0.97**
Stress P score	[AU]	3.31 ± 2.70	2.82 ± 2.36	3.93 ± 3.00 *	**0.05**	0.42
Fatigue P score	[AU]	3.37 ± 2.11	3.24 ± 1.87	3.53 ± 2.40	0.50	0.14
4SQ score	[AU]	11.89 ± 8.19	10.09 ± 6.71	14.19 ± 9.35 *	**0.02**	**0.51**

Data are expressed as mean ± SD; *p* = overall significance by independent sample *T*-Test, significant (*p* < 0.05). * vs. Male; Cohen’s d uses the sample standard deviation of the mean difference. It is not affected by the number of subjects but rather focuses on an effect’s magnitude. A value > 0.5 suggests a meaningfully medium effect, while a value >0.8 suggests a meaningfully large effect (48 spindles). Abbreviations: RR = RR interval; HR = heart rate; V = variability; RR VAR = Total Power of RR interval variability (variance); LF = low-frequency component of RR variability; a = absolute value; HF = high-frequency component of RR variability; nu= normalized unit; SAP = systolic arterial pressure; DAP = diastolic arterial pressure; ANSI = Autonomic Nervous System Index; P = perception; AU = arbitrary units; 4SQ = short somatic symptoms stress-related questionnaire. Significant values are written in bold.

**Table 2:** Individual anthropometrics and autonomic and stress perception Proxies according to playing position, considering all soccer players.

Variable		Goalkeepers	Defenders	Midfielders	Forwards	*p*
N		10	39	42	26	
Age	[yrs]	19.57 ± 4.04	19.22 ± 3.85	21.30 ± 5.14	20.20 ± 3.59	0.651
Weight	[kg]	78.10 ± 11.28	70.32 ± 11.50	66.17 ± 10.51 *	66.42 ± 11.60 *	**0.014**
Height	[cm]	184 ± 0.10	177 ± 0.11 *	173 ± 0.10 *	174 ± 0.11 *	**0.019**
BMI	[kg/m^2^]	25.20 ± 1.87	25.35 ± 1.89	25.37 ± 2.26	24.81 ± 2.20	0.824
SAP	[mmHg]	117.50 ± 14.19	119.99 ± 15.10	121.52 ± 18.66	117.08 ± 13.44	0.701
DAP	[mmHg]	68.60 ± 9.70	66.74 ± 9.25	68.76 ± 9.07	68.00 ± 8.95	0.788
HR	[beat/min]	60.21 ± 10.67	55.78 ± 10.27	54.74 ± 10.25	58.59 ± 10.25	0.291
RR	[ms]	1026 ± 191	1110 ± 201	1132 ± 205	1054 ± 181	0.258
RR VAR	[ms^2^]	5759 ± 6806	8511 ± 7488	9110 ± 8422	10,078 ± 22,319	0.826
RR LFa	[ms^2^]	1315 ± 1284	1896 ± 1802	2035 ± 1779	2247 ± 3197	0.697
RR HFa	[ms^2^]	2352 ± 3640	3864 ± 4990	4296 ± 5692	4709 ± 14,611	0.887
RR LFnu	[nu]	43.67 ± 21.42	33.21 ± 17.91	34.59 ± 15.93	43.49 ± 16.36	0.053
RR HFnu	[nu]	50.28 ± 22.53	60.58 ± 19.91	60.56 ± 18.33	50.24 ± 17.46	0.068
RR LF/HF		1.61 ± 1.95	1.01 ± 2.34	0.78 ± 0.85	1.27 ± 1.48	0.470
ANSI	[%]	44.77 ± 29.51	64.37 ± 27.09	62.15 ± 28.33	52.87 ± 26.53	0.121
Stress P score	[AU]	3.22 ± 2.68	2.69 ± 2.28	3.83 ± 3.08	3.38 ± 2.58	0.384
Fatigue P score	[AU]	4.56 ± 2.01	2.78 ± 1.93 *	3.83 ± 2.26	2.95 ± 1.88	**0.046**
4SQ score	[AU]	11.67 ± 5.66	9.56 ± 7.02	13.39 ± 9.15	12.95 ± 8.69	0.247

Data are expressed as mean ± SD; *p* = overall significance by 1WAYANOVA, followed by individual contrasts. Significant (*p* < 0.05) individual contrasts: * vs. goalkeepers; Abbreviations: RR = RR interval; HR = heart rate; V = variability; RR VAR = Total Power of RR interval variability (variance); LF = low-frequency component of RR variability; a = absolute value; HF = high-frequency component of RR variability; nu= normalized unit; SAP = systolic arterial pressure; DAP = diastolic arterial pressure; ANSI = Autonomic Nervous System Index; P = perception; AU = arbitrary units; 4SQ = short somatic symptoms stress-related questionnaire. Significant values are written in bold.

**Table 3:** Individual anthropometrics and autonomic and stress perception Proxies according to playing position, considering male soccer players.

Variable		Goalkeepers	Defenders	Midfielders	Forwards	*p*
N		7	25	27	15	
Age	[yrs]	22.14 ± 5.6	20.00 ± 3.4	21.11 ± 3.87	20.20 ± 3.59	0.501
Weight	[kg]	84.29 ± 3.86	77.76 ± 5.78 *	72.54 ± 4.79 +*	74.13 ± 6.35 +*	**0.000**
Height	[cm]	190 ± 0.03	183 ± 0.08 *	179 ± 0.05 +*	182 ± 0.07 *	**0.001**
BMI	[kg/m^2^]	25.08 ± 1.52	25.73 ± 2.01	25.75 ± 1.76	24.93 ± 1.85	0.369
SAP	[mmHg]	121.43 ± 13.14	125.92 ± 13.62	128.41 ± 18.73	123.33 ± 13.40	0.646
DAP	[mmHg]	68.71 ± 8.46	68.96 ± 8.71	71.52 ± 7.40	71.60 ± 7.75	0.581
HR	[beat/min]	59.69 ± 12.83	53.74 ± 11.00	50.07 ± 7.36	53.93 ± 7.73	0.099
RR	[ms]	1046 ± 227	1158 ± 216	1223 ± 179	1135 ± 170	0.156
RR VAR	[ms^2^]	4107 ± 2507	9927 ± 8881	10,164 ± 9550	13,360 ± 29,269	0.624
RR LFa	[ms^2^]	948 ± 643	2006 ± 1966	2083 ± 1795	2814 ± 4086	0.411
RR HFa	[ms^2^]	1430 ± 1073	4860 ± 5983	5033 ± 6775	6592 ± 19,241	0.743
RR LFnu	[nu]	44.68 ± 24.10	30.38 ± 17.19	32.48 ± 18.35	46.38 ± 17.38 +#	**0.027**
RR HFnu	[nu]	51.92 ± 22.92	65.10 ± 19.16	64.78 ± 18.47	49.25 ± 19.18 +#	**0.033**
RR LF/HF		1.63 ± 2.15	0.63 ± 0.61	0.72 ± 0.96	1.47 ± 1.78	0.068
ANSI	[%]	47.60 ± 32.80	71.33 ± 24.95 *	75.19 ± 20.80 *	60.40 ± 27.22	**0.035**
Stress P score	[AU]	3.17 ± 2.79	3.06 ± 2.58	2.90 ± 2.45	2.00 ± 1.49	0.683
Fatigue P score	[AU]	4.83 ± 1.83	2.78 ± 1.99	3.33 ± 1.83	2.90 ± 1.37	0.115
4SQ score	[AU]	11.50 ± 5.5	9.22 ± 6.71	10.67 ± 7.37	9.60 ± 6.59	0.863

Data are expressed as mean ± SD; *p* = overall significance by 1WAYANOVA, followed by individual contrasts. Significant (*p* < 0.05) individual contrasts: * vs. goalkeepers; + vs. defenders; # vs. midfielders. Abbreviations: RR = RR interval; HR = heart rate; V = variability; RR VAR = Total Power of RR interval variability (variance); LF = low-frequency component of RR variability; a = absolute value; HF = high-frequency component of RR variability; nu = normalized unit; SAP = systolic arterial pressure; DAP = diastolic arterial pressure; ANSI = Autonomic Nervous System Index; P = perception; AU = arbitrary units; 4SQ = short somatic symptoms stress-related questionnaire. Significant values are written in bold.

## Data Availability

Data will be uploaded in Zenodo if the paper is accepted. This set of raw data will be accessible under request because it includes sensitive information. Please write your request to daniela.lucini@unimi.it.

## Abbreviations

The following abbreviations are used in this manuscript:

CAR	Cardiac autonomic regulation
HRV	Heart rate variability
N	Number
BMI	Body mass index
RR	RR interval
HR	Heart rate
V	Variability
RR VAR	Total Power of RR interval variability (variance)
LF	Low-frequency component of RR variability
an	Absolute value
HF	High-frequency component of RR variability
nu	Normalized unit
SAP	Systolic arterial pressure
DAP	Diastolic arterial pressure
ANSI	Autonomic Nervous System Index
P	Perception
AU	Arbitrary units
4SQ	Short somatic symptoms stress-related questionnaire

## References

[1] Kunz M. (2007). Big Count: 265 Million Playing Football. FIFA Mag..

[2] Wragg M. (2018). FIFA Magazine Nielsen Sport. https://nielsensports.com/wpcontent/uploads/2014/12/Nielsen_World-Football-2018-6.11.18.pdf.

[3] Bull F.C., Al-Ansari S.S., Biddle S., Borodulin K., Buman M.P., Cardon G., Carty C., Chaput J.P., Chastin S., Chou R. (2020). World Health Organization 2020 Guidelines on Physical Activity and Sedentary Behaviour. Br. J. Sports Med..

[4] Visseren F., Mach F., Smulders Y.M., Carballo D., Koskinas K.C., Bäck M., Benetos A., Biffi A., Boavida J.M., Capodanno D. (2021). 2021 ESC Guidelines on Cardiovascular Disease Prevention in Clinical Practice. Eur. Heart J..

[5] Joyner M.J., Green D.J. (2009). Exercise Protects the Cardiovascular System: Effects beyond Traditional Risk Factors. J. Physiol..

[6] Lucini D., Luconi E., Giovanelli L., Marano G., Bernardelli G., Guidetti R., Morello E., Cribellati S., Brambilla M.M., Biganzoli E.M. (2024). Assessing Lifestyle in a Large Cohort of Undergraduate Students: Significance of Stress, Exercise and Nutrition. Nutrients.

[7] Mitchell J.J., Bu F., Fancourt D., Steptoe A., Bone J.K. (2022). Longitudinal Associations between Physical Activity and Other Health Behaviours during the COVID-19 Pandemic: A Fixed Effects Analysis. Sci. Rep..

[8] Douwes R., Metselaar J., Pijnenborg G.H.M., Boonstra N. (2023). Well-Being of Students in Higher Education: The Importance of a Student Perspective. Cogent Educ..

[9] Ligibel J.A., Bohlke K., May A.M., Clinton S.K., Demark-Wahnefried W., Gilchrist S.C., Irwin M.L., Late M., Mansfield S., Marshall T.F. (2022). Exercise, Diet, and Weight Management during Cancer Treatment: ASCO Guideline. J. Clin. Oncol..

[10] Linee Guida e Raccomandazioni. https://www.epicentro.iss.it/attivita_fisica/linee-indirizzo-2021.

[11] Gielen S., Schuler G., Adams V. (2010). Cardiovascular Effects of Exercise Training: Molecular Mechanisms. Circulation.

[12] Grassi G., Mark A., Esler M. (2015). The Sympathetic Nervous System Alterations in Human Hypertension. Circ. Res..

[13] El-Tahlawi M. (2021). How Do You Prevent “Sudden Death” during Sports Activities?. e-J. Cardiol. Pract..

[14] Pelliccia A., Sharma S., Gati S., Bäck M., Börjesson M., Caselli S., Collet J.P., Corrado D., Drezner J.A., Halle M. (2021). 2020 ESC Guidelines on Sports Cardiology and Exercise in Patients with Cardiovascular Disease. Eur. Heart J..

[15] Fanous Y., Dorian P. (2019). The Prevention and Management of Sudden Cardiac Arrest in Athletes. CMAJ.

[16] Finocchiaro G., Westaby J., Sheppard M.N., Papadakis M., Sharma S. (2024). Sudden Cardiac Death in Young Athletes: JACC State-of-the-Art Review. J. Am. Coll. Cardiol..

[17] Sala R., Malacarne M., Solaro N., Pagani M., Lucini D. (2017). A Composite Autonomic Index as Unitary Metric for Heart Rate Variability: A Proof of Concept. Eur. J. Clin. Investig..

[18] Lucini D., Marchetti I., Spataro A., Malacarne M., Benzi M., Tamorri S., Sala R., Pagani M. (2017). Heart Rate Variability to Monitor Performance in Elite Athletes: Criticalities and Avoidable Pitfalls. Int. J. Cardiol..

[19] Sala R., Malacarne M., Tosi F., Benzi M., Solaro N., Tamorri S., Spataro A., Pagani M., Lucini D. (2017). May a Unitary Autonomic Index Help Assess Autonomic Cardiac Regulation in Elite Athletes? Preliminary Observations on the National Italian Olympic Committee Team. J. Sports Med. Phys. Fitness.

[20] Solaro N., Malacarne M., Pagani M., Lucini D. (2019). Cardiac Baroreflex, HRV, and Statistics: An Interdisciplinary Approach in Hypertension. Front. Physiol..

[21] Lucini D., Malacarne M., Gatzemeier W., Pagani M. (2020). A Simple Home-Based Lifestyle Intervention Program to Improve Cardiac Autonomic Regulation in Patients with Increased Cardiometabolic Risk. Sustainability.

[22] Lucini D., Galiuto L., Malacarne M., Meucci M.C., Pagani M. (2021). Cardiac Autonomic Effects of Yearly Athletic Retreats on Elite Basket Players: Usefulness of a Unitary Autonomic Nervous System Indicator. Sustainability.

[23] Lucini D., Fallanca A., Malacarne M., Casasco M., Galiuto L., Pigozzi F., Galanti G., Pagani M. (2020). Streamlining Analysis of RR Interval Variability in Elite Soccer Players: Preliminary Experience with a Composite Indicator of Cardiac Autonomic Regulation. Int. J. Environ. Res. Public Health.

[24] Solaro N., Pagani M., Lucini D. (2021). Altered Cardiac Autonomic Regulation in Overweight and Obese Subjects: The Role of Age-and-Gender-Adjusted Statistical Indicators of Heart Rate Variability and Cardiac Baroreflex. Front. Physiol..

[25] Lucini D., Spataro A., Giovanelli L., Malacarne M., Spada R., Parati G., Solaro N., Pagani M. (2022). Relationship between Body Composition and Cardiac Autonomic Regulation in a Large Population of Italian Olympic Athletes. J. Pers. Med..

[26] Lucini D., Sala R., Spataro A., Malacarne M., Benzi M., Tamorri S., Pagani M. (2018). Can the Use of a Single Integrated Unitary Autonomic Index Provide Early Clues for Eventual Eligibility for Olympic Games?. Eur. J. Appl. Physiol..

[27] McEwen B.S., Akil H. (2020). Revisiting the Stress Concept: Implications for Affective Disorders. J. Neurosci..

[28] Henningsen P., Zipfel S., Herzog W. (2007). Management of Functional Somatic Syndromes. Lancet.

[29] Kivimäki M., Pentti J., Ferrie J.E., Batty G.D., Nyberg S.T., Jokela M., Virtanen M., Alfredsson L., Dragano N., Fransson E.I. (2018). Work Stress and Risk of Death in Men and Women with and without Cardiometabolic Disease: A Multicohort Study. Lancet Diabetes Endocrinol..

[30] Bart R., Ishak W.W., Ganjian S., Jaffer K.Y., Abdelmesseh M., Hanna S., Gohar Y., Azar G., Vanle B., Dang J. (2018). The Assessment and Measurement of Wellness in the Clinical Medical Setting: A Systematic Review. Innov. Clin. Neurosci..

[31] Herbert C. (2022). Enhancing Mental Health, Well-Being and Active Lifestyles of University Students by Means of Physical Activity and Exercise Research Programs. Front. Public Health.

[32] Lazarus R.S. (2000). How Emotions Influence Performance in Competitive Sports. Sport. Psychol..

[33] Song Z., Baicker K. (2019). Effect of a Workplace Wellness Program on Employee Health and Economic Outcomes: A Randomized Clinical Trial. JAMA.

[34] Schnermann M.E., Schulz C.A., Ludwig C., Alexy U., Nöthlings U. (2022). A Lifestyle Score in Childhood and Adolescence Was Positively Associated with Subsequently Measured Fluid Intelligence in the DONALD Cohort Study. Eur. J. Nutr..

[35] Bosquet L., Merkari S., Arvisais D., Aubert A.E. (2008). Is Heart Rate a Convenient Tool to Monitor Over-Reaching? A Systematic Review of the Literature. Br. J. Sports Med..

[36] Krishna Bandi H., Keerthi S.G., Reddy M.N., Singh M.S.B. (2012). Spectral Analysis of Short Term Heart Rate Variability in Healthy Volunteers: The Role of Gender. Int. J. Sci. Res. Publ..

[37] Lucini D., Solaro N., Lesma A., Gillet V.B., Pagani M. (2011). Health Promotion in the Workplace: Assessing Stress and Lifestyle with an Intranet Tool. J. Med. Internet Res..

[38] Lucini D., Riva S., Pizzinelli P., Pagani M. (2007). Stress Management at the Worksite: Reversal of Symptoms Profile and Cardiovascular Dysregulation. Hypertension.

[39] Fritz C.O., Morris P.E., Richler J.J. (2012). Effect Size Estimates: Current Use, Calculations, and Interpretation. J. Exp. Psychol. Gen..

[40] Coote J.H. (2013). Myths and Realities of the Cardiac Vagus. J. Physiol..

[41] Plews D.J., Laursen P.B., Stanley J., Kilding A.E., Buchheit M. (2013). Training Adaptation and Heart Rate Variability in Elite Endurance Athletes: Opening the Door to Effective Monitoring. Sports Med..

[42] White D.W., Raven P.B. (2014). Autonomic Neural Control of Heart Rate during Dynamic Exercise: Revisited. J. Physiol..

[43] Smith D.L., Horn G.P., Petruzzello S.J., Freund G.G., Bloom S.I., Fernhall B. (2022). Hemostatic Responses to Multiple Bouts of Firefighting Activity: Female vs. Male Differences in a High Demand, High Performance Occupation. Int. J. Environ. Res. Public Health.

[44] Strath S.J., Kaminsky L.A., Ainsworth B.E., Ekelund U., Freedson P.S., Gary R.A., Richardson C.R., Smith D.T., Swartz A.M. (2013). Guide to the Assessment of Physical Activity: Clinical and Research Applications: A Scientific Statement from the American Heart Association. Circulation.

[45] Manzi V., Iellamo F., Impellizzeri F., D’Ottavio S., Castagna C. (2009). Relation between Individualized Training Impulses and Performance in Distance Runners. Med. Sci. Sports Exerc..

[46] Iellamo F., Legramante J.M., Pigozzi F., Spataro A., Norbiato G., Lucini D., Pagani M. (2002). Conversion from Vagal to Sympathetic Predominance with Strenuous Training in High-Performance World Class Athletes. Circulation.

[47] Kivimäki M., Steptoe A. (2018). Effects of Stress on the Development and Progression of Cardiovascular Disease. Nat. Rev. Cardiol..

[48] Purvis D., Gonsalves S., Deuster P.A. (2010). Physiological and Psychological Fatigue in Extreme Conditions: Overtraining and Elite Athletes. Phys. Med. Rehabil..

[49] Mirto M., Filipas L., Altini M., Codella R., Meloni A. (2024). Heart Rate Variability in Professional and Semiprofessional Soccer: A Scoping Review. Scand. J. Med. Sci. Sports.

[50] Hayano J., Yuda E. (2019). Pitfalls of Assessment of Autonomic Function by Heart Rate Variability. J. Physiol. Anthropol..

[51] Beissner F., Meissner K., Bär K.J., Napadow V. (2013). The Autonomic Brain: An Activation Likelihood Estimation Meta-Analysis for Central Processing of Autonomic Function. J. Neurosci..

[52] Pagani M., Lombardi F., Guzzetti S., Rimoldi O., Furlan R., Pizzinelli P., Sandrone G., Malfatto G., Dell’Orto S., Piccaluga E. (1986). Power Spectral Analysis of Heart Rate and Arterial Pressure Variabilities as a Marker of Sympatho-Vagal Interaction in Man and Conscious Dog. Circ. Res..

[53] Hess W.R. (1949). Nobel Lecture. Nobel Lectures, Physiology or Medicine (1942–1962).

[54] Malik M., Camm A.J., Bigger J.T., Breithardt G., Cerutti S., Cohen R.J., Coumel P., Fallen E.L., Kennedy H.L., Kleiger R.E. (1996). Heart Rate Variability. Standards of Measurement, Physiological Interpretation, and Clinical Use. Eur. Heart J..

[55] Saleem S.H.M. (2012). Gender Differences of Heart Rate Variability in Healthy Volunteers. J. Pak. Med. Assoc..

[56] Reimann M., Friedrich C., Gasch J., Reichmann H., Rüdiger H., Ziemssen T. (2010). Trigonometric Regressive Spectral Analysis Reliably Maps Dynamic Changes in Baroreflex Sensitivity and Autonomic Tone: The Effect of Gender and Age. PLoS ONE.

[57] Fu Q. (2023). Sex Differences in Autonomic Function. Primer on the Autonomic Nervous System.

[58] McQuilliam S.J., Clark D.R., Erskine R.M., Brownlee T.E. (2022). Mind the Gap! A Survey Comparing Current Strength Training Methods Used in Men’s versus Women’s First Team and Academy Soccer. Sci. Med. Footb..

[59] Baumgart C., Hoppe M.W., Freiwald J. (2014). Different Endurance Characteristics of Female and Male German Soccer Players. Biol. Sport..

[60] McFadden B.A., Walker A.J., Bozzini B.N., Sanders D.J., Arent S.M. (2020). Comparison of Internal and External Training Loads in Male and Female Collegiate Soccer Players During Practices vs. Games. J. Strength. Cond. Res..

[61] Burfeind K., Hong J., Stavrianeas S. (2012). Gender Differences in the Neuromuscular Fitness Profiles of NCAA Division III Soccer Players. Isokinet. Exerc. Sci..

[62] Nunome H., Drust B., Dawson B. (2013). Science and Football VII: The Proceedings of the Seventh World Congress on Science and Football. Science and Football VII: The Proceedings of the Seventh World Congress on Science and Football.

[63] Hunter S.K., Angadi S.S., Bhargava A., Harper J., Hirschberg A.L., Levine B.D., Moreau K.L., Nokoff N.J., Stachenfeld N.S., Bermon S. (2023). The Biological Basis of Sex Differences in Athletic Performance: Consensus Statement for the American College of Sports Medicine. Med. Sci. Sports Exerc..

[64] Mujika I., Santisteban J., Impellizzeri F.M., Castagna C. (2009). Fitness Determinants of Success in Men’s and Women’s Football. J. Sports Sci..

[65] J Helgerud J.H.U.W. (2013). Gender Differences in Strength and Endurance of Elite Soccer Players. Science and Football IV.

[66] Gioldasis A. (2017). Technical Skills According to Playing Position of Male and Female Soccer Players. Int. J. Sci. Cult. Sport.

[67] Garnica-Caparrós M., Memmert D. (2021). Understanding Gender Differences in Professional European Football through Machine Learning Interpretability and Match Actions Data. Sci. Rep..

[68] Pappalardo L., Rossi A., Natilli M., Cintia P. (2021). Explaining the Difference between Men’s and Women’s Football. PLoS ONE.

[69] Gullett N., Zajkowska Z., Walsh A., Harper R., Mondelli V. (2023). Heart Rate Variability (HRV) as a Way to Understand Associations between the Autonomic Nervous System (ANS) and Affective States: A Critical Review of the Literature. Int. J. Psychophysiol..

[70] Maron B.J., Pelliccia A. (2006). The Heart of Trained Athletes: Cardiac Remodeling and the Risks of Sports, Including Sudden Death. Circulation.

[71] Lochbaum M., Stoner E., Hefner T., Cooper S., Lane A.M., Terry P.C. (2022). Sport Psychology and Performance Meta-Analyses: A Systematic Review of the Literature. PLoS ONE.

[72] Leblanc V.R. (2009). The Effects of Acute Stress on Performance: Implications for Health Professions Education. Acad. Med..

[73] La Rovere M.T., Gorini A., Schwartz P.J. (2022). Stress, the Autonomic Nervous System, and Sudden Death. Auton. Neurosci..

[74] Sheppard M.N. (2012). Aetiology of Sudden Cardiac Death in Sport: A Histopathologist’s Perspective. Br. J. Sports Med..

[75] Putukian M. (2016). The Psychological Response to Injury in Student Athletes: A Narrative Review with a Focus on Mental Health. Br. J. Sports Med..

[76] Gustafsson H., Sagar S.S., Stenling A. (2017). Fear of Failure, Psychological Stress, and Burnout among Adolescent Athletes Competing in High Level Sport. Scand. J. Med. Sci. Sports.

[77] Nicholls A.R., Gustafsson H., Gerber M., Nixdorf I., Beckmann J., Nixdorf R. (2020). Psychological Predictors for Depression and Burnout Among German Junior Elite Athletes. Front. Psychol..

[78] Kim H.G., Cheon E.J., Bai D.S., Lee Y.H., Koo B.H. (2018). Stress and Heart Rate Variability: A Meta-Analysis and Review of the Literature. Psychiatry Investig..

[79] Pagani E., Gavazzoni N., Bernardelli G., Malacarne M., Solaro N., Giusti E., Castelnuovo G., Volpi P., Carimati G., Lucini D. (2023). Psychological Intervention Based on Mental Relaxation to Manage Stress in Female Junior Elite Soccer Team: Improvement in Cardiac Autonomic Control, Perception of Stress and Overall Health. Int. J. Environ. Res. Public Health.

[80] Krygier J.R., Heathers J.A.J., Shahrestani S., Abbott M., Gross J.J., Kemp A.H. (2013). Mindfulness Meditation, Well-Being, and Heart Rate Variability: A Preliminary Investigation into the Impact of Intensive Vipassana Meditation. Int. J. Psychophysiol..

[81] Brown L., Rando A.A., Eichel K., Van Dam N.T., Celano C.M., Huffman J.C., Morris M.E. (2021). The Effects of Mindfulness and Meditation on Vagally Mediated Heart Rate Variability: A Meta-Analysis. Psychosom. Med..

[82] Russo M.A., Santarelli D.M., O’Rourke D. (2017). The Physiological Effects of Slow Breathing in the Healthy Human. Breathe.

[83] Kirk U., Axelsen J.L. (2020). Heart Rate Variability Is Enhanced during Mindfulness Practice: A Randomized Controlled Trial Involving a 10-Day Online-Based Mindfulness Intervention. PLoS ONE.

[84] Bernardi L., Sleight P., Bandinelli G., Cencetti S., Fattorini L., Wdowczyc-Szulc J., Lagi A. (2001). Effect of Rosary Prayer and Yoga Mantras on Autonomic Cardiovascular Rhythms: Comparative Study. BMJ.

[85] Iellamo D., Pigozzi F., Spataro A. (2006). Autonomic and Psychological Adaptations in Olympic Rowers. J. Sports Med. Phys. Fit..

[86] Button K.S., Ioannidis J.P.A., Mokrysz C., Nosek B.A., Flint J., Robinson E.S.J., Munafò M.R. (2013). Power Failure: Why Small Sample Size Undermines the Reliability of Neuroscience. Nat. Rev. Neurosci..

